# Autologous platelet-rich gel versus vitamin B12 for the treatment of grade III-IV acute radiation dermatitis in malignant tumor patients undergoing radiotherapy: an open-label randomized controlled clinical trial

**DOI:** 10.3389/fonc.2025.1576458

**Published:** 2025-11-07

**Authors:** Haiyun Tao, Chun Xiong, Lanju Tan, Na Xie, Jiaxin Chen, Huadong Xie, Wenjun Le, Hengcheng Zhong, Weiming Liang, Yongqi Shen

**Affiliations:** 1 Department of Tumor Hematology, the First Affiliated Hospital of Guangxi University of Science and Technology, Liuzhou, Guangxi, China; 2 First Clinical Medical College, Guangxi University of Science and Technology,, Liuzhou, Guangxi, China; 3 Department of Radiotherapy, People’s Hospital of Guangxi Zhuang Autonomous Region, Nanning, Guangxi, China

**Keywords:** acute radiation dermatitis, autologous platelet rich gel, radiation therapy, quality of life instrument for cancer patients in china, malignant tumors

## Abstract

**Background:**

Acute radiation-induced dermatitis (ARD) remains the most prevalent adverse event associated with radiotherapy;however, effective management options remain limited.This study was designed to evaluate the efficacy and safety of autologous platelet-rich gel(APRG) versus vitamin B12 in the treatment of grade III to IV acute radiation dermatitis in patients with malignant tumors.

**Materials and methods:**

40 patients diagnosed with Grade III–IV acute ARD were randomly assigned in a 1:1 ratio to either the APRG group (n = 20) or the vitamin B12 group (n = 20). All patients received wound cleansing prior to intervention. In the APRG group, the prepared gel was sprayed onto the wound surface, which was then covered with sterile Vaseline gauze and a secondary dressing for 2 to 3 days. In the vitamin B12 group, wounds were treated with a wet compress consisting of 5 mg vitamin B12 dissolved in 100 mL normal saline, applied for 30 minutes per session, three times daily. ARD severity was graded according to the RTOG criteria, and adverse events (AEs) were assessed using CTCAE v4.0 by trained oncologists. The numerical rating scale (NRS) for the topical pain and Quality of Life Instrument for Cancer Patients in China (QOL-CCC) were subjectively reported by patients.

**Results:**

The APRG group demonstrated a significant shortening in the median healing time for ARD compared to the Vitamin B12 group (3.0 days, IQR: 2.0-4.0 vs. 8.0 days, IQR: 5.0-12.0; p < 0.001). Furthermore, patients treated with APRG reported consistently and significantly lower pain levels from day 2 through day 7 post-therapy (F = 24.288, P < 0.001). Superior improvements in five key quality-of-life domains—sleep, mental state, fatigue, activities of daily living, and appetite—were also observed in the APRG group (all P < 0.05). Critically, no treatment-related adverse events were recorded in the APRG group during the follow-up period.

**Conclusion:**

Our findings establish APRG as a superior and transformative intervention over Vitamin B12, demonstrating significant efficacy in accelerating wound healing, reducing pain, improving patient quality of life, and maintaining a safe treatment profile.

**Registration Details:**

https://www.chictr.org.cn/showproj.html?proj=222262, identifier ChiCTR2400081656.

## 1 Introduction

Radiation therapy or radiotherapy (RT) is currently one of the most widely used treatment options for cancer around the world (1); approximately 50% of patients undergo radiation treatment during the course of their disease.which is painful owing to erosions and affects the patient’s quality of life. Radiation dermatitis is a common side effect of radiotherapy (RT) in cancer patients (2); with 60%–95% of patients develop this condition and 85% exhibiting moderate to severe skin reactions (3).

Acute and chronic manifestations of radiation-induced skin damage include burning, pruritus, pain, hyperpigmentation, dry or moist desquamation, and erythema, while severe cases may present with edema, ulceration, hemorrhage, necrosis, and local infection (4).ARD typically manifests within 90 days following the initiation of radiotherapy (5), which causes pain, wound infection, and poor quality of life. (6).The severity of ARD varies depending on treatment-related factors (e.g., radiation dose, irradiated volume, bolus, concurrent chemotherapy, treatment positioning, etc.) and intrinsic factors (e.g., body mass index, irradiation site, smoking status, and skin pigmentation) (7).Severe cases may necessitate treatment interruption, consequently reducing tumor control rates and overall survival time (8).

Significant strides have been made in refining RT techniques to improve their precision and minimize side effects.Contemporary advancements such as stereotactic body radiation therapy (SBRT), intensity-modulated radiation therapy, hypofractionated radiation therapy, and partial breast irradiation have notably enhanced treatment efficacy and reduced skin toxicity (9). Yet, the prevalence of radiodermatitis remains high among certain patient populations (10). No criterion standard currently exists for the treatment of acute radiation-induced ARD (11). While certain agents have demonstrated efficacy in managing radiation dermatitis, large-scale, multicenter randomized controlled trials(RCT) are lacking, resulting in inconsistent outcomes across existing prevention and management strategies (4). This represents a significant challenge for clinicians, underscoring the need to investigate more effective therapeutic approaches (12, 13).Platelet-rich plasma (PRP) is a rich source of growth factors, such as platelet-derived growth factor, epithelial growth factor, and vascular endothelial growth factor, which directly contribute to skin repair.

The purpose of this study is to determine the efficacy and safety of autologous platelet-rich gel in the treatment of Grade III-IV acute radiation dermatitis in cancer patients.

## 2 Materials and methods

For requirements for a specific article type please refer to the Article Types on any Frontiers journal page. Please also refer to Author Guidelines for further information on how to organize your manuscript in the required sections or their equivalents for your field^1^.

### 2.1 Ethical approval and consent

The study was carried out in accordance with the Declaration of Helsinki and the International Conference on Harmonisation Tripartite Guideline on Good Clinical Practice. Prior to participating, all patients furnished signed informed consent. The Ethics Committee of the First Affiliated Hospital of Guangxi University of Science and Technology granted approvals in March 2024 (approval number: 2024-LC3). The study was registered with the Chinese Clinical Trial Registry (ChiCTR2400081656).

### 2.2 Patients

Inclusion criteria: Eligible patients were required to have: (1) a confirmed malignant tumor diagnosis via histology; (2) developed Grade III-IV acute radiation dermatitis following radiotherapy; (3) intact communicative and cognitive abilities; and (4) provided voluntary written informed consent.

Exclusion criteria:(1) tumor-related ulceration; (2) psychiatric or intellectual impairment impairing normal verbal communication; (3) history of multiple drug allergies or recent allergic disorders; (4) severe cardiopulmonary comorbidities (including unstable angina, class II cardiac insufficiency, acute myocardial infarction, acute exacerbation of chronic obstructive pulmonary disease, or pulmonale).

### 2.3 Methods and design

This study is an open-label randomized clinical trial,which was conducted at the First Affiliated Hospital of Guangxi University of Science and Technology in China. 40 patients with malignant tumors who developed grade III-IV ARD after radiotherapy were enrolled in this study from March 2024 to August 2025.In this study, All patients were assigned sequential inclusion numbers.40 patients were randomly assigned in a 1:1 ratio to either the APRG group (n = 20) or the vitamin B12 group (n = 20).All patients underwent wound cleansing prior to treatment.Vitamin B12 Group:5mg of vitamin B12 dissolved in 100ml of normal saline was applied to the wound as a wet compress for 30 minutes each time, three times daily.APRG Group: The prepared Autologous Platelet Rich Gel (APRG) was sprayed onto the surface of the wound. Subsequently, sterile petroleum jelly gauze and sterile gauze were placed over the wound to provide coverage for a duration of 2 to 3 days.

### 2.4 Preparation method for autologous platelet-rich gel

1. (1) The ratio of the volume of venous blood drawn (ml) to the area of radiation dermatitis (cm²) is 1:10, as determined by the extent of the patient’s radiation dermatitis.
2. The withdrawn peripheral venous blood was transferred into anticoagulant tubes containing ethylenediaminetetraacetic acid (EDTA) and subsequently centrifuged at 4°C at a speed of 2000 revolutions per minute (rpm) for a duration of 4 minutes.
3. On an ultra-clean table, the upper layer of plasma and the platelet fraction were carefully aspirated and transferred to EDTA anticoagulant tubes. These tubes were subsequently centrifuged at 4 °C at a speed of 4000 rpm for a duration of 6 minutes.
4. After centrifugation, the supernatant is collected using an ultra-clean workbench, while the lowest layer, which contains platelet-rich plasma (PRP), is PRP, which is moved to an EDTA tube and mixed thoroughly.
5. An activator was synthesized by combining thrombin with calcium gluconate at a concentration of 1000 U/mL.
6. The platelet-rich gel was prepared using a 10:1 volume ratio of Au-PRP and the activator (14).

### 2.5 Outcome measurements

#### 2.5.1 Primary outcome measurement

The primary outcome of this study was the APRG healing time (days). ARD Healing Assessment:The primary endpoint of ARD healing time was defined as the duration from randomization until the affected skin area improved to Grade 1 or achieved complete re?epithelialization. ARD severity was evaluated daily during treatment using the Radiation Therapy Oncology Group (RTOG) scoring criteria (12), and the time to healing was documented accordingly.The ARD level was reported using the Radiation Therapy Oncology Group (RTOG)criteria: grade 0, no change; grade 1, follicular, faint, or dull erythema or dry desquamation; grade 2, tender or bright erythema or patchy moist desquamation; grade 3, confluent, moist desquamation other than skin folds; and grade 4, ulceration, hemorrhage, or necrosis (13).

#### 2.5.2 Secondary outcome measurements

Secondary outcomes included changes in pain NRS scores, QOL-CCC scores, and adverse event incidence.

Based on preliminary observations, APRG-treated patients exhibited pain relief and wound healing initiation within 2 days, whereas the vitamin B12 group showed a comparable response only after 7 days. Efficacy and safety evaluations were therefore conducted at baseline (D0), day 2 (D2), and day 7 (D7).The numerical rating scale (NRS) for the topical pain and Quality of Life Instrument for Cancer Patients in China (QOL-CCC) were subjectively reported by patients.Adverse effects(AE) were assessed using the CTCAE v4.0 by trained oncologists.

Pain Intensity: Patient-reported pain due to ARD was quantified using the NRS, an 11-point scale ranging from 0 (no pain) to 10 (worst imaginable pain), with higher scores indicating greater pain severity (15).

Quality of Life: The Quality of Life Instrument for Cancer Patients in China (QOL-CCC) was administered to evaluate five key domains: appetite, sleep, mental status, fatigue, and activities of daily living. Each domain is scored on a 5-point Likert-type scale, where 1 represents the poorest and 5 the best possible status (16).

Safety Profile: All adverse events observed during the treatment period were systematically recorded by trained oncology physicians and nurses.

### 2.6 Statistical analysis

All data are expressed as mean ± standard deviation, interquartile range, or numbers (%).Normally distributed data between the two groups were assessed using the Shapiro-Wilk test, and variables at different time points within each group were compared by repeated measure ANOVA. Continuous variables with non-normal distributions were compared between the two groups using the Wilcoxon rank sum test. Categorical data were compared using the Χ^2^ test. All statistical analyses were performed with IBM SPSS version 26.0 statistical software. A p-value of < 0.05 were defined as statistically significant.

## 3 Result

### 3.1 Study sample and baseline characteristics

**Figure 1:**
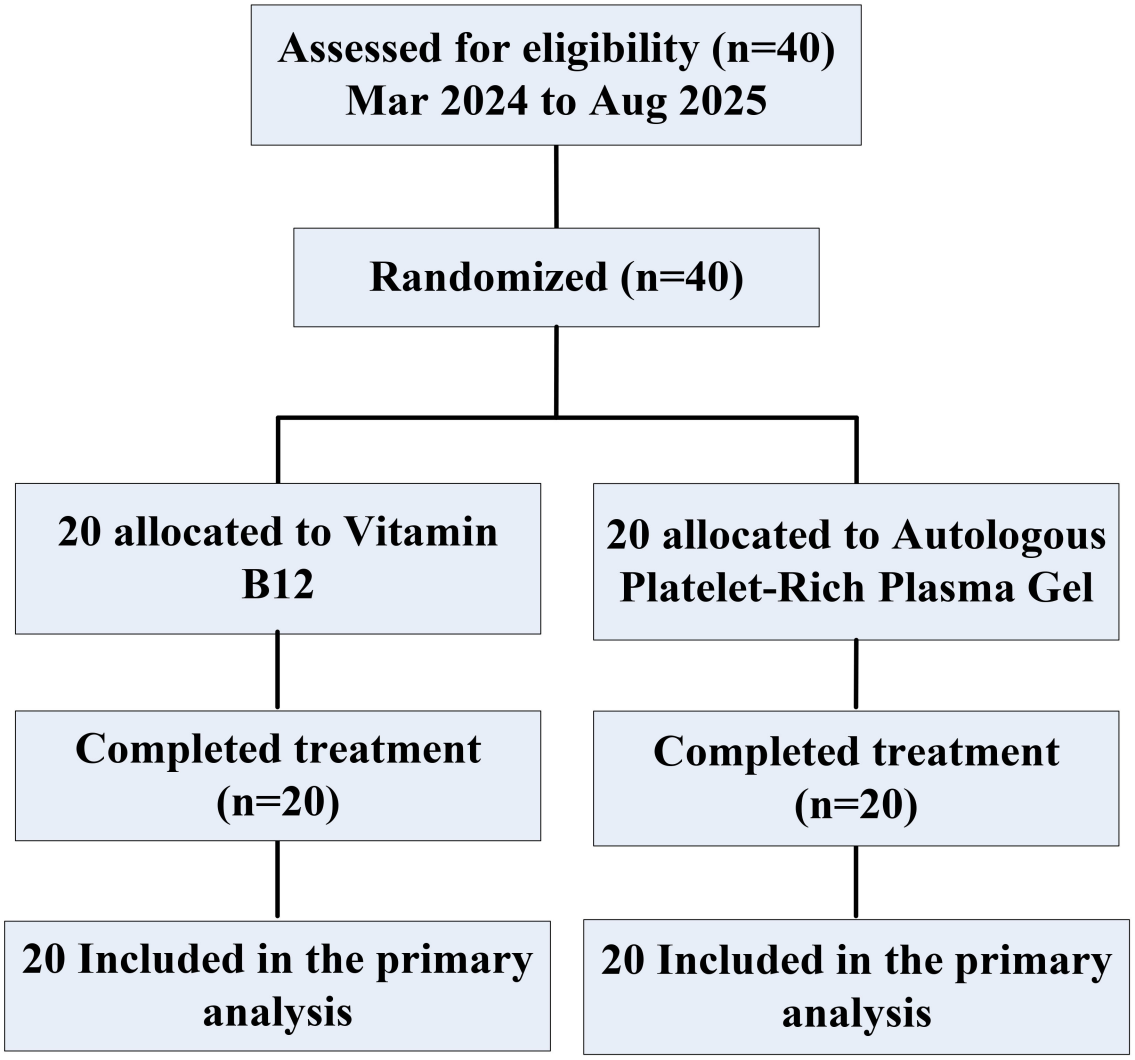
CONSOR diagram.

Between March 2024 and August 2025, 40 eligible patients with grade III–IV ARD were identified acros the First Affiliated Hospital of Guangxi University of Science and Technology,and were randomly assigned to the the Autologous Platelet-Rich Plasma gel (APRG) group or the vitamin B12 group. All 40 patients (20 in the APRG group and 20 in the vitamin B12 group) were included in the primary analysis. Every participant completed all study-related procedures (**Figure 1**).

The baseline patient characteristics were balanced between the APRG group and the vitamin B12 group (**Table 1**),The average patient age was 59 years (range, 45–73 years) and 60% of the patients were female.Of these patients, 52.5% had breast cancer, and 42.5% had Head and Neck Cancer(HNC), and 5% had Cervical Cancer.The distribution of radiation dermatitis was 45% Grade III and 50% Grade IV.57.5% of patients received adjuvant radiotherapy,and 42.5% received concurrent chemoradiotherapy.

**Table 1:** Characteristics of two patient groups.

	Vitamin B12 group (n=20)	APRG Group (n=20)	*P*
Gender, n (%)			
Male	7(35)	9(45)	0.519
Female	13(65)	11(55)	
Age, median (range), y	58(45–73)	59(49–72)	
<65	16(80)	15(75)	0.456
≥65	4(20)	5(25)	
ECOG, n (%)			
1	13 (65)	12(60)	0.744
2	7 (35)	8 (40)	
Origin of tumor, n (%)			
Head and neck cancer	8(40)	9(45)	0.948
Breast cancer	11(55)	10(50)	
Cervical Cancer	1(5)	1(5)	
EORTC, n (%)			
III	10(50)	8(40)	0.525
IV	10(50)	12(60)	
treatment, n (%)			
Adjuvant Radiotherapy	11(55)	12(60)	0.342
Concurrent Chemoradiotherapy	9(45)	8(40)	

### 3.2 ARD Healing Time

The Shapiro-Wilk test indicated that ARD healing time in the APRG group deviated from a normal distribution (p < 0.001). Consequently, a Mann-Whitney U test was employed to compare the groups. The median ARD healing time was significantly shorter in the APRG group (3.0 days, IQR: 2.0-4.0) than in the Vitamin B12 group (8.0 days, IQR: 5.0-12.0), U = -5.45, p < 0.001 (**Table 2**).

**Table 2:** Comparison of ARD healing time between study groups.

Group	N	ARD healing time(Days) median(lQR)	Z	*P*
Vitamin B12 Group	20	8.0(IQR: 6.0-9.8)	-5.45	< 0.001
APRG Group	20	3(IQR: 2.0-4.0)		

The median ARD healing time was significantly shorter in the APRG group (3.0 days, IQR: 2.0-4.0) than in the Vitamin B12 group (8.0 days, IQR: 5.0-12.0), U = -5.45, p < 0.001.

### 3.3 NRS Scores by Study Group

**Figure 2:**
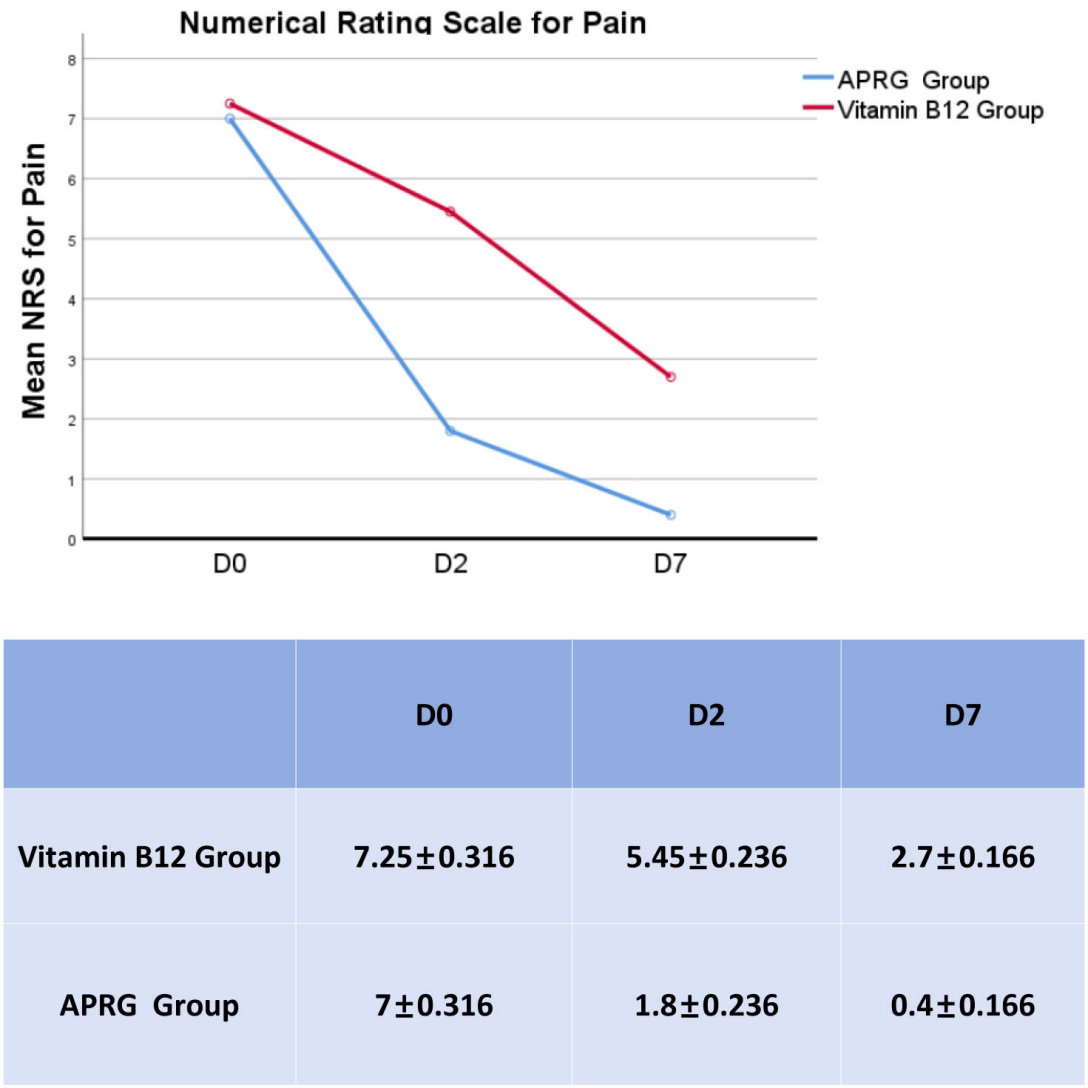
Comparison of mean NRS scores between study groups. NRS scores for pain at three evaluation points:pre-therapy (D0), post-therapy day 2(D2), and post-therapy day 7(D7).The APRG group demonstrated consistently and significantly lower pain levels than the Vitamin B12 group from day 2 post-therapy(F=24.288,P<0.001).

Numerical rating scale (NRS) scores for pain at three evaluation points:pre-therapy(D0), post-therapy day 2(D2), and post-therapy day 7(D7). The APRG group demonstrated consistently and significantly lower pain levels than the Vitamin B12 group from day 2 post-therapy(F=24.288, P<0.001) (**Figure 2**).

### 3.4 Changes in quality of life before and after treatment

**Figure 3:**
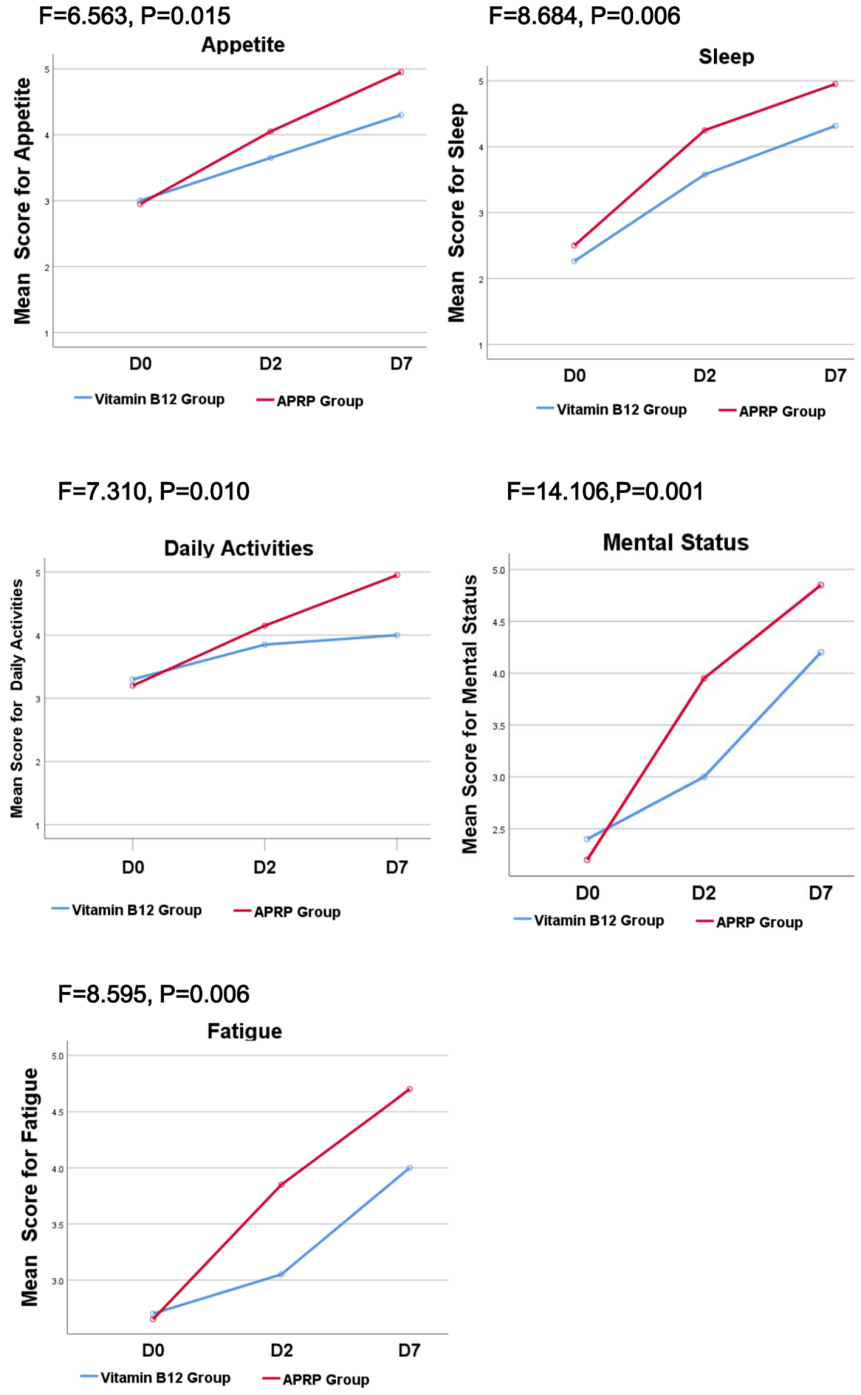
QOL-CCC between the vitamin B12 group and APRG group. Patient quality of life was assessed using the Quality of Life Instrument for Cancer Patients in China (QOL-CCC). Compared to the vitamin B12 group, patients receiving APRG therapy demonstrated statistically significant superior improvements in five key domains: sleep, mental state, fatigue, activities of daily living, and appetite (P < 0.05).

Patient quality of life was assessed using the Quality of Life Instrument for Cancer Patients in China (QOL-CCC). Compared to the vitamin B12 group, patients receiving APRG therapy demonstrated statistically significant superior improvements in five key domains: sleep, mental state, fatigue, activities of daily living, and appetite (P < 0.05).(**Figure 3**).

### 3.5 Adverse events

In the APRG group, two cases developed localized pruritus at 2 days after topical application, with symptoms resolving promptly upon removal of the dressing. No APRG-related adverse events were observed in any patient during the follow-up period.

## 4 Discussion

In this open-label, randomized clinical trial, we investigated the efficacy and safety of APRG versus vitamin B12 in the management of ARD. The results demonstrated that APRG offers significantly superior clinical value compared to the conventional vitamin B12 regimen. Our comprehensive assessment of wound healing, pain control, quality of life, and safety profiles provides compelling evidence to support the clinical application of APRG.

Radiation dermatitis represents a frequent and clinically significant complication of radiotherapy (3). This condition not only causes substantial physical discomfort and psychological distress, but also markedly compromises patients’ quality of life (17). In severe cases, it may lead to systemic infections and unplanned treatment interruptions, ultimately adversely affecting tumor control rates (18). Hence, developing rapidly effective strategies for managing radiation dermatitis remains a crucial priority in clinical oncology. Currently, robust evidence supporting definitive efficacy of any particular agent or intervention for alleviating radiation dermatitis is lacking. While several international associations have proposed relevant clinical guidelines, no definitive consensus has been established in this area (19).

A universally accepted standard of care for radiation dermatitis has yet to be established (20). Current clinical approaches to radiation dermatitis encompass both preventive and therapeutic strategies: (1) Proactive prevention through technological advances: The implementation of modern radiotherapy techniques, including three-dimensional conformal radiotherapy and intensity-modulated radiotherapy, has demonstrated efficacy in minimizing radiation-induced skin toxicity. (9). (2) General preventive measures: ① Wear loose absorbent cotton clothes, avoid rubbing and scratching the skin; ② Keep the skin clean, wash with warm water, avoid using irritating toiletries; ③Keep the irradiated field skin dry and avoid sunlight; ④It is recommended to eat a high-calorie, high-protein, high-vitamin diet, drink more water, and avoid spicy and stimulating food (21). (3) Existing treatment methods: ① Hyperbaric oxygen therapy (HBOT): Studies have shown that HBOT applied to patients’ skin lesions can effectively increase the oxygen supply function of skin lesions, reduce the inflammatory exudation of wounds, and accelerate the dryness and healing of wounds (22); ② Vitamin B12: Current studies at home and abroad believe that vitamin B12 mixed solution can not only promote the healing of radiation dermatitis, reduce pain, but also reduce the occurrence of radiation dermatitis (23); ③. Steroid hormones: corticosteroids can inhibit the proliferation of radioactive cytokines, so they are often used to treat radiation dermatitis (24). Many studies have shown that topical corticosteroids can reduce the incidence of radiation dermatitis (25), relieve pain and skin itching (26); ④ Calendula: The role of calendula in radiation dermatitis is controversial, with studies (24) suggesting that topical use of calendula can promote the risk of the development of radiation dermatitis, while Fabiana Verdan Simoes believes (27) that calendula has great potential in the prevention and treatment of radiation dermatitis; ⑤ Tranexamic acid: Amanda Rosenthal (4) found that treatment with plasminogen inhibitor tranatemocyclic acid can reduce the incidence of radiation dermatitis in plg +/+ mice, and inhibiting radiation dermatitis in plg +/- mice and inhibiting plasminogen activation can be a new therapeutic strategy to reduce and prevent the occurrence of radiation dermatitis in patients.These treatments have shown some effectiveness, but they are not capable of rapidly and effectively controlling radiation dermatitis within a short period, and their therapeutic effects are limited. Therefore, there is a need for us to explore new treatment methods.

Platelet-rich plasma (PRP) is a concentrate of autologous or allogeneic whole blood that is prepared after the collection of whole blood and contains a high concentration of platelets. Depending on the source of the whole blood, it can be classified into autologous platelet-rich plasma (Au-PRP) and allogeneic platelet-rich plasma (Al-PRP) (28). APRG is a product of Au-PRP activated by calcium chloride and bovine thrombin, containing various growth factors that are beneficial for cell proliferation (29). Its efficacy in the treatment of diabetic foot ulcers is well- established (30), and PRP has also been very successful in many other fields. Au-PRP has been gradually applied in neurosurgery, orthopedics and oral repair in clinical practice. It does not increase the incidence of systemic or wound adverse reactions (26), but there are only basic studies on the treatment of radiation dermatitis. Lee et al. studied the regenerative function of PRP by locally irradiating the dorsal skin of mice and found that Au-PRP could enhance cell function through the AKT signaling pathway, thereby promoting the regeneration of damaged skin. They believe that the capacity of PRP to promote skin healing warrants clinical research and application (31).

In the study of Tao Yilei et al,the healing time of radiation dermatitis treated with vitamin B12 was 9.1 days on average (32), while the healing time of pearl layer powder combined with vitamin B12 was 7–16 days, with an average of 9.5 days (33). In this study, the most pivotal finding is that APRG significantly shortens the median healing time of ARD. The healing time in the APRG group was only 3.0 days, compared to 8.0 days in the Vitamin B12 group, a difference that was statistically significant (p < 0.001). Rapid wound healing is crucial for alleviating patient discomfort, reducing the risk of secondary infection, and ensuring the uninterrupted progression of radiotherapy.

The management of ARD-associated pain is challenging and frequently necessitates high-dose opioid analgesics, which are often accompanied by significant side effects.In terms of pain reduction, a meta-analysis of the effects of topical corticosteroids in preventing radiation dermatitis found that topical corticosteroids did not reduce the pain caused by radiotherapy (4). There are no reports of pain alleviation in clinical studies combining vitamin B12 for the treatment of radiation dermatitis (21, 23). However, in this study,the APRG treatment group demonstrated superior efficacy in pain relief. From day 2 to day 7 post-treatment, the APRG group exhibited a consistent and statistically significant reduction in pain levels compared to the Vitamin B12 group (P < 0.001). The prompt analgesic effect of APRG can be attributed to a confluence of mechanisms: swift re-epithelialization shielding exposed nerve endings, a favorable shift in the inflammatory landscape mediated by cytokines like IL-1Ra, and the creation of a moist, protective wound milieu that fosters healing.

Radiation dermatitis significantly compromises the quality of life in cancer patients. An open-label, randomized controlled trial demonstrated that xenogeneic PRP emulsion effectively prevents and delays the onset of acute radiation dermatitis, showing significantly superior efficacy in improving patient quality of life compared to XONRID^®^ gel (18). Notably, our study utilizing APRG observed similarly promising outcomes. The results indicate that APRG significantly outperforms vitamin B12 in enhancing patient quality of life, demonstrating multidimensional benefits. These advantages were consistently observed across five key domains: sleep quality, mental status, fatigue levels, activities of daily living, and appetite. The observed superiority of APRG is likely attributable to significantly shortened median healing time and sustained pain relief in the APRG group, which collectively establish a solid foundation for comprehensive quality of life improvement.

significantly improving patients’ sleep, mental state, fatigue, daily living activities, and appetite, thereby enhancing the patients’ quality of life. In this study, only two cases of skin itching were observed, and symptoms were relieved after the removal of the oil gauze, which is considered to be caused by the oil gauze itself, indicating good safety.

The results of this study indicate that autologous platelet-rich gel is rapid, safe, effective and can significantly improve the quality of life of patients with grade III-IV acute radiation dermatitis.

Study limitations, This study has several limitations, including its single-center design and relatively limited sample size, which may affect the generalizability of the findings. Future large-scale, multicenter randomized controlled trials are required to validate our conclusions. Further investigation into the mechanism of action of APRG in ARD treatment, its potential synergistic effects with advanced wound dressings, and the identification of predictive biomarkers for treatment response represent promising directions for future research.

In summary, our findings indicate that compared to vitamin B12, APRG demonstrates potential advantages in promoting ARD healing, providing sustained analgesia, and improving quality of life, while exhibiting favorable tolerability in the studied population. These data suggest APRG as a promising comprehensive strategy for ARD management. The observed clinical benefits are likely attributable to its rich bioactive composition, which warrants further investigation to elucidate the underlying mechanisms and optimize its therapeutic application.

## Data Availability

The datasets presented in this study can be found in online repositories. The names of the repository/repositories and accession number(s) can be found in the article/supplementary material.

## Footnotes

1 For Original Research articles, please note that the Material and Methods section can be placed in any of the following ways: before Results, before Discussion or after Discussion.
